# The Walnut’s Dark Secret: Polyphenol Oxidase and the Enigmatic Pathway to Melanin

**DOI:** 10.3390/ijms27041681

**Published:** 2026-02-09

**Authors:** Abhaya M. Dandekar, Noah G. Feinberg, Paulo A. Zaini, Ramona Abbattista, Renata de Almeida Barbosa Assis, Sriema L. Walawage

**Affiliations:** Department of Plant Sciences, University of California Davis, 1 Shields Ave., Davis, CA 95616, USA; ngfeinberg@ucdavis.edu (N.G.F.); pazaini@ucdavis.edu (P.A.Z.); rabbattista@ucdavis.edu (R.A.); slwalawage@ucdavis.edu (S.L.W.)

**Keywords:** melanin biosynthesis, polyphenol oxidase (PPO), walnut (*Juglans regia*), enzymatic darkening, plant defense, oxidative stress, redox homeostasis, metabolic evolution

## Abstract

The biosynthesis of melanin in plants remains an enduring biochemical enigma. Unlike the well-characterized pathways of animals and fungi that produce the canonical “true melanins”, the enzymatic origins and physiological functions of melanin-like pigments in plants are poorly described. Recent advances in *Juglans regia* (walnut) have begun to illuminate this “dark metabolism,” revealing a dual polyphenol oxidase (PPO) system, constitutive *JrPPO1* and stress-inducible *JrPPO2*, that orchestrates the oxidation of phenolics into amorphous, heterogeneous polymeric pigments. Functional studies demonstrate that *JrPPO1* maintains tyrosine and redox homeostasis, while silencing triggers a lesion-mimic phenotype, highlighting the enzyme’s role in detoxifying reactive intermediates. In contrast, *JrPPO2* responds to redox and pathogen stress, driving pigment formation as part of the defense response. The integration of biological evidence, encompassing genomics, genetics, and phenotyping, reveals that walnut pigmentation represents a genetically encoded, developmentally regulated adaptation balancing metabolic cost and oxidative protection. Decoding this system reframes melanin biosynthesis in plants as a strategic redox resilience mechanism, one that transforms potentially toxic phenolic oxidation into protective polymerization, bridging primary metabolism, defense, and evolution.

## 1. Introduction

Melanin is one of nature’s most ancient and ubiquitous pigments, present in all kingdoms of life, with critical functions from absorbing UV radiation, scavenging reactive oxygen species (ROS) to its contribution to structural resilience and immune defenses [1,2]. In animals and microorganisms, the biosynthesis of true melanins primarily takes place with the oxidation and polymerization of tyrosine derived intermediates such as dopaquinone, leading to the formation of eumelanin and pheomelanin biopolymers. In plants, however, melanin biosynthesis remains an enigma. Despite the widespread occurrence of black and brown pigments, particularly after wounding, infection, or in tissues developmentally regulated such as seed coats, the molecular identity of plant “melanin”, its biosynthetic route, physiological role(s), and distinction from other similarly colored pigments remain obscure [3,4]. Unlike animals and fungi, plants lack a canonical tyrosinase-based pathway, and the enzymes and substrates responsible for melanin-like pigment formation are only partially defined. This ambiguity has fueled the longstanding view that melanogenesis in plants is merely a byproduct of oxidative phenolic degradation by way of enzymatic browning, a notion now being challenged by recent molecular and biochemical evidence [5].

Among the enzymes implicated, polyphenol oxidases (PPOs) occupy center stage. PPOs are copper-containing metalloenzymes that can catalyze the cresolase and/or catecholase-type two-electron oxidation of phenolic compounds to highly reactive quinones, which subsequently polymerize into dark pigments resembling melanin [3,4,6]. PPOs are ancient and widespread, yet their physiological purpose remains elusive. Interestingly, they are absent in the well-studied model plant system *Arabidopsis thaliana* but are very prominent in crop plants like apple, potato, tomato, and walnut among others, where their activity correlates with color changes during development, postharvest storage and/or defense [4,5,6]. This striking variability points to fundamental and unresolved questions: What is the true function of melanin biosynthesis in plants? Why has it evolved so divergently across lineages?

Walnut (*Juglans regia*) offers a useful and powerful model to address this mystery. The walnut genome encodes two distinct PPO isoforms, *JrPPO1* (Gene ID: 109020781) and *JrPPO2* (Gene ID: 108979139), each with specialized expression patterns and biological properties which together weave a complex narrative linking melanin formation to both defense and development [7,8]. The story deepens with *JrPPO1*, which performs an unexpected, constitutive role in tyrosine hydroxylation within living, normally functioning tissues [9,10,11,12]. Silencing *JrPPO1* in walnut triggers a dramatic lesion mimic phenotype, revealing a central role of PPO in maintaining cellular integrity [5]. The loss of *JrPPO1* activity results in the accumulation of toxic intermediates such as tyramine and phenolic amines, uncontrollable oxidation, and spontaneous cell death [5]. Simultaneously, *JrPPO2* operates in the shadows, its presence is induced under oxidative or pathogen stress, particularly in roots and wounded tissues and drives the formation of dark pigments under stress conditions [8]. Walnut provides a uniquely tractable system to investigate this problem; not because it is exceptional, but because it makes an otherwise cryptic pathway experimentally visible. Most plant species contain polyphenol oxidases and accumulate suitable phenolic substrates for fueling oxidative polymerization; yet, in many annual model systems, these processes remain latent, transient, or developmentally restricted. In *Juglans regia*, the high basal PPO activity, the clear spatial segregation of constitutive and inducible isoforms, and the pronounced accumulation of dark pigments in defined contexts expose the core architecture of a pathway that is likely conserved but ambiguous in other plants. The walnut system therefore acts as a magnifying lens, revealing mechanistic principles that apply broadly to plant phenolic oxidation, redox regulation, and stress adaptation. The discovery of this dual PPO system was first revealed by the *J. regia* genome sequence and has since reshaped our understanding of how plants regulate phenol oxidation and pigment formation at the molecular level [7,9]. Taken together, these collective findings position walnut’s biosynthesis of melanin-like polymers as a metabolic trade-off between defense and energy allocation, a sophisticated adaptation evolved to protect reproductive tissues and maintain metabolic stability under oxidative stress.

Thus, while the chemical composition of plant “melanin” may vary among species depending on available substrates, the unifying principle of enzyme-initiated oxidation of phenolics leading to protective polymer formation is likely shared across diverse taxa. Walnut offers a rare combination of genetic resolution, biochemical tractability, and visible phenotypic output needed to dissect this pathway in detail, providing insights that extend to oxidative pigmentation, defense chemistry, and redox buffering mechanisms throughout the plant kingdom. Ultimately, decoding the “dark secret” of walnuts’ melanin-like pigments promises to bridge a long-standing gap in plant biochemistry, transforming our perception of a pigment once dismissed as a byproduct into a key regulator of plant survival and adaptation.

## 2. Melanin: The Enduring Enigma of Plant Biochemistry

### 2.1. True Melanins: The Hallmarks of the Animal and Microbial Kingdoms

As previously stated, melanin is one of nature’s oldest, most versatile, and widespread biological pigments that spans the kingdoms of life. Between absorbing UV radiation, scavenging reactive oxygen species (ROS), contributing to cellular structural resilience, and aiding in immune defense, melanin covers a wide range of essential biological roles [1,2]. But what do we mean when we say “melanin”?

Melanin, or more accurately melanins, refers to a broad category of high molecular weight biopolymers consisting of multiple classes and subclasses. Classifications have changed over time and vary depending on the delineating factor: primarily biochemical origin and biogenic process. From a biochemical origin perspective, the three classes of melanins are the indolic melanins (subclasses: eumelanins, pheomelanins, and neuromelanins), the allomelanins (subclasses: phenolic melanins, DHN melanins), and the pyomelanins. Though they vary from each other, these classes may be considered “true melanins” due to a shared set of chemical and physical properties: they are heterogeneous, amorphous polymers formed through quinone-mediated oxidative polymerization, and are characterized by broadband UV–Vis absorbance, persistent radicals, potent redox activity, high capacity for metal chelation, and exceptional chemical stability and recalcitrance. Due to their relevance to human appearance and health, it is the indolic melanin subclasses that have received the greatest attention. However, the plant and microbially derived allomelanins and microbial pyomelanins, which possess similarly impressive arrays of interesting chemical and physical attributes, have increasingly become the focus of contemporary melanin research.

Notably, while melanins are generally thought of as being brown–black in color, this is not a requirement nor a qualifier for the title of “true melanin”. It so happens that the indolic eumelanins and neuromelanins, as well as many of the allomelanins, confer brown–black pigmentation, but this is not the case for the yellow to red pheomelanins or the pyomelanins, which range in color from dark brown to reddish-brown. Recognition of this fact is important, if not essential, in delineating melanins from other biological pigments: many of which confer similar coloration in the brown–black spectrum. This is particularly the case in plants, which possess many distinct non-melanin pigments such as proanthocyanidins and hydrolysable tannins, as well as color-imparting structural polymers like lignins: which all share color attributes but not biochemical origin.

### 2.2. Plant Melanins: Enigmatic Functional Analogs of the Canonical Melanins

The long-standing enigma in plant biochemistry is put simply: do plants make true melanins? While there is no shortage of brown and black pigments in the plant biochemical arsenal, the question of whether these are true melanins remains a hotly debated topic.

Brown and black pigmentation in plants has historically been associated with physical wounding, infection by pathogens, senescence, and rotting. The darkening that occurs during these processes is the uncontrolled oxidative process of “enzymatic browning” (EB), where damaged and/or infected tissues of plant cells facilitates the oxidation and polymerization of vacuole-localized phenolic compounds by plastid-localized phenolic oxidases [13]. These resulting melanin-like polymers meet the definition of a true melanin; however, a few key differences distinguish them from their animal and microbial counterparts. First is the substantial difference in substrate utilization, as plants produce a much wider array of phenolic compounds that become incorporated into these melanin-like polymers. These include amino acids (like tyrosine), hydroxycinnamic and hydroxybenzoic acids, and flavonoids, among others. This produces polymers that lack the distinct subunit structures of the true melanins and are likely complex matrices of oxidized phenolics crosslinked through C–O and C–C bonds, stabilized by metal chelation and hydrogen bonding. Secondly, this process is less coordinated than the melanogenic processes of other organisms, and functions more like the triggering of a well-hidden trap when circumstances demand rapid and potent oxidative defense.

Set apart from the process of EB is the developmental accumulation of melanin-like polymers in the living tissues of various plant species, especially within seed coats. The study of these pigments is often complicated by the co-occurrence of other plant-derived brown and black pigments, in addition to the natural compositional and structural complexity that occurs from oxidative polymerization reactions. Based on the chemical and spectroscopic properties of these pigments, their melanic nature has been confirmed in the seed coats of a variety of species including tomato, black mustard, barley, watermelon, and others [4]. Accumulation of melanin-like pigments have also been reported in persimmon, where the polymers are deposited on the cell walls of cells in the upper epidermal and subepidermal layers of the fruit skin [14]. Further insights gained through combinatorial application of ultraviolet-visible wavelength (UV–Vis) spectroscopy, electron paramagnetic resonance (EPR) spectroscopy, matrix-assisted laser desorption/ionization-time of flight mass spectrometry (MALDI-TOF MS), and Fourier transform infrared (FTIR) spectroscopy revealed the melanic pigment in oat to be a p-coumaric acid-based homopolymer [15]. While it is not known if the composition of other melanin-like polymers is similarly uniform, this homogeneity suggests an alternative biosynthetic route compared to the more haphazard and random products of enzymatic browning.

### 2.3. The Paradox of Plant “Melanogenesis”

As for whether or not these two types of plant-derived melanin-like polymers are truly melanins, or are simply melanin-like, the reality remains that they serve as functional analogs to the true melanins present in the other kingdoms of life. And this brings us to the enduring paradox of plant “melanogenesis”. On one hand, enzyme-mediated oxidation and pigment deposition are hallmarks of oxidative defense, providing antimicrobial protection and structural reinforcement. On the other, excessive pigment production consumes valuable metabolites and can compromise photosynthesis, respiration, and energy homeostasis along with other essential primary metabolic processes. Every molecule diverted toward melanin-like polymerization represents a metabolic cost: consumption of carbon skeletons, oxygen, and reducing power that could otherwise sustain growth. This tension between protection and productivity defines the ‘melanin paradox’ in plants. The repeated evolution and retention of this chemical hallmark across plant lineages therefore implies that the energetic investment in melanogenesis yields critical adaptive benefits, chiefly the stabilization of redox homeostasis and long-term tissue protection under stress. And among the many genetic and metabolic players entwined with this process, one group stands above the rest: the polyphenol oxidases.

## 3. Polyphenol Oxidases: Catalysts at the Crossroads of Phenol Metabolism

### 3.1. Structure and Catalytic Mechanisms of PPOs

**Figure 1:**
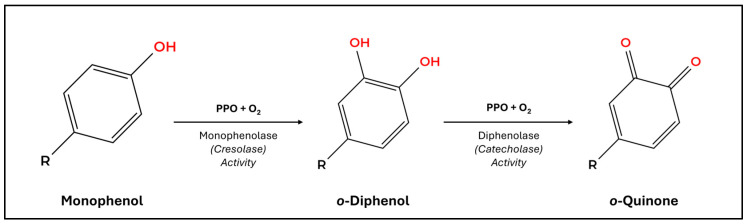
Overview of polyphenol oxidase (PPO)-mediated oxidation of phenolic compounds. Monophenolase, or cresolase, activity facilitates the *ortho*-hydroxylation of monophenols into diphenols using molecular oxygen. Subsequent oxidation of *ortho*-diphenols into reactive *ortho*-quinones is achieved by diphenolase, or catecholase, activity along with molecular oxygen. The resulting *o*-quinones react with other phenolic compounds to form amorphous, heterogeneous “melanin-like” polymers.

Polyphenol oxidases (PPOs) are copper-containing metalloenzymes belonging to the type-3 copper protein family, which also includes tyrosinases and catechol oxidases. Their active site consists of a dinuclear copper-binding center (CuA and CuB) coordinated by six conserved histidines, enabling molecular oxygen to accept electrons from phenolic substrates. PPOs catalyze two distinct but related reactions that mirror the enzymatic chemistry of true melanogenesis: (i) monophenolase or cresolase activity, hydroxylating monophenols such as p-tyrosine to o-diphenols (e.g., DOPA); (ii) diphenolase or catecholase activity, oxidizing o-diphenols to o-quinones (Figure 1). These quinones are highly reactive electrophiles that spontaneously polymerize or crosslink with amino acids, yielding dark brown–black-colored polymers. As previously discussed, these are the melanin-like pigments produced in plants.

In plants, PPOs are synthesized as pre-pro-enzymes with twin N-terminal transit peptides directing them to the chloroplast through the inner and outer membrane and directing them to the chloroplast thylakoid membranes or plastoglobuli, where the latent form is sequestered until activation by proteolytic cleavage or membrane disruption. This tight spatial control prevents uncontrolled oxidation of phenolics during normal metabolism, which is largely synthesized in the cytosol. Upon wounding or stress, exposure of PPO to phenolic substrates initiates localized oxidation, pigment formation, and crosslinking of cell wall proteins, contributing to both mechanical and chemical defense. This admixture of enzyme and substrate are brought into contact in the case of physical wounding or membrane leakage during stress, though the precise mechanisms facilitating enzyme-substrate interactions in intact, living cells remain largely undescribed.

It has been long speculated that PPOs play a more essential role in chloroplast function of living, intact cells based on their localization near the photosynthetic machinery [16]. The ability for PPOs to utilize phenolic compounds as sinks for excess reducing power during plastid stress is one explanation, but it begs the question: what are these luminal-facing, thylakoid-bound enzymes using as substrate? A limited number of investigations have begun to explore chloroplast phenolic metabolism to address this question. Findings in red clover have revealed plausible plastid-localized substrates for PPOs. Comparison of normal and light-stressed leaves revealed the apparent PPO-mediated conversion of the monophenolic coumaroyl malate to the diphenolic caffeoyl malate, without any noted conversion of the diphenol into a melanin-like pigment [17]. These results, while limited in scope, suggest that PPOs may have access to suitable substrates for managing reducing power in the plastid. And while not co-localized to the same sub-plastid compartment, the shikimic acid pathway (which notably produces tyrosine) is localized to the plastid stroma. However, more work in this area is needed to determine the presence of thylakoid-localized substrates, or a means by which stroma-localized substrates encounter the thylakoid-bound PPOs.

### 3.2. Distribution and Functional Diversity in the Plant Kingdom

Although PPO genes are found in nearly all plant lineages, their copy number, expression pattern and physiological roles vary dramatically. Some species, such as *Arabidopsis thaliana*, lack functional PPO genes entirely, whereas others, including apple, potato, avocado, and walnut, harbor large, divergent PPO families. This uneven distribution underscores the lineage-specific evolution of PPO-dependent oxidative metabolism. In solanaceous species, PPOs participate in defense against herbivory and pathogen infection, while in cereals, legumes, and tree crops, their activity is associated with seed coat pigmentation and postharvest discoloration. The functional redundancy and context dependent activation of these enzymes have historically obscured their true physiological purpose. Alongside their association with undesirable commercial quality traits, the perception of PPOs has been reinforced as simply “browning enzymes” rather than regulators of specialized metabolism.

Recent phylogenetic analysis indicates that plant PPOs have undergone repeated duplication and neofunctionalization, giving rise to isoforms with distinct substrate specificities and regulatory controls [7,9,12]. In several woody perennials, including *Juglans*, these duplications appear to coincide with adaptations to oxidative stress and long-life cycles, suggesting an evolutionary pressure to balance defense, redox homeostasis, and phenolic turnover [9,18].

### 3.3. PPOs as Metabolic Integrators

Beyond their catalytic chemistry, PPOs function as quantitative regulators of phenolic flux, redox balance, and stress metabolism. In walnut, this role is supported by multiple independent measurements showing that PPO activity directly determines the magnitude and direction of phenolic oxidation. Upon infection with *Xanthomonas arboricola* pv. *juglandis*, PPO enzymatic activity in leaves increases approximately 1.8–2.3-fold within 24–72 h, accompanied by a parallel induction of *JrPPO1* transcripts and pathogenesis-related protein [19]. Similarly, stress-inducible *JrPPO2* expression rises by more than 3-fold under oxidative stress conditions, with a corresponding increase in catecholase activity [20]. These values demonstrate that PPO activation is not marginal but represents a major metabolic shift during defense. Quantitative metabolic consequences of PPO disruption are revealed by *JrPPO1* silencing. Araji et al. [5] reported that suppression of *JrPPO1* caused a 2–4-fold accumulation of tyramine and a significant increase in hydroxycinnamoyl–tyramine conjugates, together with elevated lipid peroxidation and visible lesion formation. These measurements establish that PPO activity normally channels a substantial fraction of aromatic amino acid metabolism away from toxic intermediates and toward stable oxidative end products.

Together, these observations indicate that PPOs function as quantitative metabolic integrators. Changes of 2–3-fold in PPO abundance or activity propagate into much larger differences in pigment accumulation and redox status. This amplification behavior is characteristic of flux-controlling nodes in metabolism and explains why PPO perturbation produces system level phenotypes such as lesion mimicry and oxidative hypersensitivity.

## 4. The Walnut Model: Decoding the Dual PPO System and the Hidden Pathway to Melanin

### 4.1. Walnut as a Model for the Study of Plant Melanin Biosynthesis

Among higher plants, *Juglans regia* stands out as a useful natural model for elucidating the enzymatic basis of plant melanogenesis. Its tissues exhibit remarkable phenolic content and polyphenol oxidase activity, particularly in sites where oxidative pigmentation, wound darkening, and pathogen defense converge. Early biochemical studies of walnut tissue darkening associated these color changes with phenol oxidation, but the molecular underpinnings remained unknown until the cloning and functional characterization of *JrPPO1* and *JrPPO2* [3].

### 4.2. Genomic Insights from Walnut: The Dual PPO System

The sequencing of the *Juglans regia* ‘Chandler’ genome revealed an unexpected duplication of the PPO locus, yielding two distinct genes, *JrPPO1* and *JrPPO2* [7,9,18]. These paralogs differ in expression profile, regulatory behavior, catalytic efficiency, and substrate specificity [8,9,12,19,20]. Broadly speaking, the two walnut PPOs can be classified as tissue-regulated and developmentally regulated, in green tissues in the case of *JrPPO1* [3,9], and stress-inducible, in the case of *JrPPO2*; this shows a 20-fold increase in expression 48 h after infection with bacterial blight disease [8].

**Figure 2:**
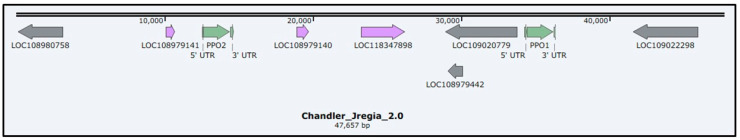
The highly conserved 47,657 bp region of *Juglans regia* chromosome 3 that contains both *JrPPO1* and *JrPPO2* along with intervening genetic loci. The coding sequence of both *JrPPO1* (615 aa) and *JrPPO2* (610 aa) were deduced from the gene sequence, as they each are a single exon containing two chloroplast transit peptides typically required for proteins that traverse both the outer and inner and the thylakoid membranes in the chloroplast and a C-terminal thylakoid membrane binding domain [7]. The two coding sequences share an overall 71.46% amino acid residue identity, it is notably, that the copper-binding domain Cu-A are identical between the copies while Cu-B possesses critical amino acid differences that have been shown to impact substrate specificity and enzyme kinetic properties [9,12].

*JrPPO1* was first cloned using mRNA acquired from female (pistillate) flowers, the earliest stage in the reproductive life cycle and production of edible walnuts [3]. The resulting sequence revealed a Type-3 copper protein with two copper-binding domains, as described by crystal structure analysis of catechol oxidase from sweet potato by Klabunde et al. [21]. Independent x-ray crystallography of *JrPPO1* confirmed this protein structure [22]. Interestingly, Southern blots revealed a single gene [3]. However, the version 1 sequence of the walnut genome revealed the presence of a second PPO gene, *JrPPO2*, on a different contig [7]. Improvement of the genomic assembly to version 1.5 that included several *Juglans* species revealed that both *JrPPO1* and *JrPPO2* were located on the same contig, and that *JrPPO1* arose from *JrPPO2* via a gene duplication event ~60 MYA [9]. The version 2 genome sequence of the walnut genome that has a mosaic chromosomal level assembly revealed that both genes are located next to each other towards one end of chromosome 3 as predicted [18]. A 47 kb region that contains *JrPPO1* and *JrPPO2* is shown in Figure 2 below.

Comparative genomic studies across *Juglans* uncovered accelerated divergence of PPO1 and conservation of PPO2 among lineages, consistent with neofunctionalization/subfunctionalization following duplication [9]. This same study revealed that the two genes have undergone accelerated sequence evolution, with specific amino acid substitutions in the copper-binding domain B likely responsible for their differences in substrate affinity and redox potential. This hypothesis was supported through site directed mutagenesis experiments of the *JrPPO1* 1st-activity controller residue (Asn240) that impaired its monophenolase activity: mimicking the natural difference in walnut PPO isoforms and effectively converting *JrPPO1* into *JrPPO2* [23]. These modifications indicate plausible pathways through selection that favored allelic variants of PPOs that optimize oxidative resilience.

The Juglandaceae-specific duplication appears to have been retained because it confers adaptative flexibility, allowing basal control of phenolic metabolism through *JrPPO1* while deploying *JrPPO2* as an inducible oxidative defense. From an evolutionary standpoint, the persistence and diversification of PPOs across *Juglans* species indicate a metabolic trade-off between defense capacity and oxidative cost: thus, explaining the functional heterogeneity and regulatory complexity observed within the walnut PPO family.

Population-level analysis further demonstrates selection signatures surrounding *PPO* loci in cultivated germplasm, linking these genes, or at least their genomic loci, to the domestication process and selected agronomic qualities [11].

The coexistence of constitutive and inducible PPO isoforms exemplifies how plants can modulate oxidative phenolic metabolites as a dynamic stress response. As we will explore in the coming sections, the “constitutive” pathway maintains homeostasis, while the inducible pathway triggers pigment formation under stress as a resolution mechanism. This modular design may represent an evolutionary resolution to the paradox of melanin biosynthesis in plants, enabling controlled oxidation without sacrificing metabolic stability.

### 4.3. Tissue-Specific and Developmental JrPPO1: Guardian of Phenolic Homeostasis

#### 4.3.1. *JrPPO1* Functionality

**Figure 3:**
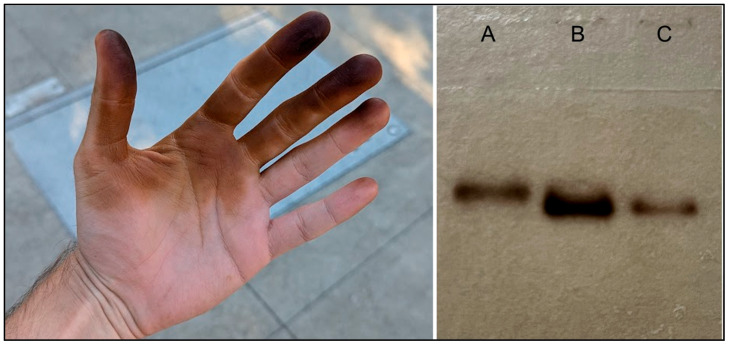
Staining of human skin through contact with wounded walnut tissues (left) and PPO activity assays from hull tissues (right). The juices from the disrupted walnut hull tissues will stain human skin (left), a hallmark of manual walnut hull removal, which is minimally removed by washing and only reverts to normal by replacement with new skin cells. The same reaction is observed on native polyacrylamide gels (right) when assaying for PPO activity. Total protein (10 μg) extracted from hull tissues is used for in-gel tyrosinase staining and was the means by which *JrPPO1* was originally discovered. Lane A shows the pure mushroom tyrosinase control, while lanes B and C show extracts from the hull epidermal tissue and cortical tissue, respectively.

Functional analysis has shown that *JrPPO1*, the first cloned PPO from walnut, is widely expressed in green tissues with notable expression in hull and pistillate flowers [3]. Proteomic evidence has also revealed the presence of *JrPPO1* in the walnut pellicle (seed coat) at developmental maturity [10]. Its enzymatic activity encompasses both cresolase and catecholase functions, enabling the hydroxylation of monophenols to diphenols, such as L-tyrosine to L-DOPA, and the subsequent oxidation of these diphenols to o-quinones, as with L-DOPA to dopaquinone [24]. This dual activity mirrors tyrosinase-like behavior and suggests that *JrPPO1* may operate analogously to canonical melanogenic enzymes, but within the context of plant phenol metabolism. Handling of damaged, fresh walnut tissues, as when manually hulling walnut fruits, demonstrates the potent activity of *JrPPO1* as a melanogenic enzyme (Figure 3).

#### 4.3.2. The Metabolic Sentinel: Lessons from *JrPPO1* Silencing Experiments

##### The Lesion Mimic Phenotype

Silencing of *JrPPO1* in transgenic walnut through RNAi exposed an unanticipated and dramatic outcome, “the lesion mimic phenotype”, in which spontaneous necrotic spots and premature senescence appeared on otherwise uninfected tissues [5]. These lesions mirrored a hypersensitive-response-like pattern; with localized cell death surrounded by chlorotic halos, suggesting that *JrPPO1* activity normally restrains rather than promotes oxidative injury. The phenotype was accompanied by an increase in lipid peroxidation, depletion of antioxidant capacity, and accumulation of phenolic amines such as tyramine: collectively indicating that the absence of *JrPPO1* disrupts intracellular redox equilibrium. The accumulation of biogenic amines is particularly noteworthy, as the exogenous application of tyramine on walnut explants reproduced the lesion phenotype and triggered cell death [5]. Taken together, the observation that simple suppression of a single enzyme can elicit such profound metabolic impacts demonstrated that *JrPPO1* sits at a critical metabolic juncture.

##### Tyrosine Metabolism, Redox Homeostasis, and Programmed Cell Death

Detailed metabolic profiling and transcriptomic analysis of *JrPPO1*-silenced plants revealed the essential role that *JrPPO1* plays in orchestrating the health and wellness of the plant cell [5].

Metabolomic analysis revealed a rerouting of tyrosine metabolism away from oxidative deamination toward amine conjugation, resulting in high levels of tyramine and hydroxycinnamoyl-tyramine derivatives [5]. These short-lived compounds generally appear in stressed plant tissues as a part of the defense response: providing antimicrobial properties, redox buffering capacity, and cell wall reinforcement in the apoplast. However, excessive accumulation of these molecules without proper management can lead to disruption of growth and development, as well as cell death (as evidenced by exogenous application experiments in walnut). While not responsible for their biosynthesis, the disruption of *JrPPO1* functionality drastically increased and sustained the levels of these potent biomolecules in silenced walnut lines [5]. While *JrPPO1* is known to hydroxylate tyrosine to L-DOPA, this evidence further suggests that it may play an essential role in converting tyramine to dopamine; thereby limiting the accumulation of tyramine and its derivatives [5]. Thus, *JrPPO1*’s catalytic function serves a necessary and protective role, converting hazardous byproducts of amino acid metabolism into stable and potentially beneficial end products.

Transcriptomic analyses of the same *JrPPO1*-silenced tissues revealed the upregulation of defense-related and cell-death-associated genes, including pathogenesis-related proteins and components of the salicylic acid signaling pathway [5]. These molecular signatures parallel those observed during pathogen-induced hypersensitive responses, implying that loss of *JrPPO1* functionality and the downstream consequences mimic biotic stress at the signaling level [5]. In conjunction with disruptions to tyramine detoxification, the ensuing cell death may be triggered by ROS-dependent activation of MAP–kinase cascades and by accumulation of quinonoid electrophiles that covalently modify nucleophilic residues in key enzymes [5].

#### 4.3.3. Conceptual Implications for *JrPPO1*

The *JrPPO1*-silencing studies transform the interpretation of plant PPOs from merely “browning” enzymes into essential metabolic safety mechanisms [5]. While the widely expressed “constitutive” *JrPPO1* has the biosynthetic capacity for melanogenesis and is likely the key enzyme driving wound-induced browning, these aforementioned findings indicate a necessary reframing. In normally functioning tissues, *JrPPO1* acts not as a mere browning enzyme but as a critical metabolic regulator at the intersection of primary and secondary metabolism, governing the flux between amino acid utilization, phenolic turnover, and cell viability in non-melanized tissues [5].

### 4.4. Inducible JrPPO2: The Stress-Responsive Oxidase

#### 4.4.1. *JrPPO2* Functionality

The discovery of *JrPPO2* provided a second dimension of walnuts oxidative metabolism. Unlike *JrPPO1*, *JrPPO2* expression is inducible, particularly under oxidative stress, pathogen attack or wounding [8,20,25,26]. It is strongly expressed in roots and tissues undergoing defense responses [8,9]. When expressed heterologously, *JrPPO2* exhibits robust catecholase activity toward a wide range of diphenols, generating dark polymers even under low substrate concentrations. *In planta*, its induction coincides with enhanced expression of ROS-detoxifying enzymes and pathogenesis-related proteins, suggesting a coordinated response network. Taken together, this evidence is consistent with *JrPPO2* being a stress-activated pigment forming enzyme.

#### 4.4.2. Walnut’s “Dark Metabolism”: *JrPPO2*-Mediated Melanogenesis for Defense

##### The Role of PPOs in the War Against Pathogens

In the plant kingdom, oxidative enzymes are central players in the first line of defense. Upon wounding or pathogen attack, plants mount an ‘oxidative burst’, releasing reactive oxygen species (ROS) and activating enzymes that reinforce cell walls or detoxify radicals. Within this oxidative milieu, PPOs serve as both catalysts and regulators. PPO activity complements the NADPH oxidase-driven generation of ROS. The quinones produced by PPOs can react with hydrogen peroxide and superoxide radicals, forming semi-quinone radicals that both scavenge and propagate oxidative signals. In this way, PPOs function as amplifiers and modulators of redox signaling, shaping the intensity and duration of the defense response.

The spatial localization of PPOs further refines this control. In uninfected tissues, the enzymes are confined to plastids; upon attack, cell wall disruption and vesicular trafficking release active PPO into the cytosol and apoplast, where phenolic substrates accumulate. The resulting oxidative microenvironment promotes localized polymerization without causing systemic damage. This precise targeting distinguishes controlled defense pigmentation from uncontrolled necrosis observed in PPO-silenced lines (Section 4.3).

##### The Walnut Immune Arsenal: *JrPPO2* Upregulation During Pathogenesis

In walnut, infection by *Xanthomonas arboricola* pv. *juglandis* (Xaj), the causal agent of bacterial blight, induces a rapid and localized increase in PPO activity. Khodadadi et al. [19] demonstrated that *JrPPO1* transcripts and total PPO enzyme activity more than doubled within 24 h post-infection, paralleling the upregulation of the pathogenesis-related gene *P14a*. This response coincides with visible tissue darkening at infection sites, consistent with in situ oxidation of phenolic substrates and deposition of melanin-like polymers that physically encapsulate invading bacteria. However, this was before the discovery of *JrPPO2*, and subsequent investigations into the host response to bacterial blight revealed *JrPPO2* to be the pathogen-induced copy responsible for driving the characteristic pigmentation in infected walnut tissues [8]. This is consistent with promoter analyses of the two walnut PPO genes, which revealed the *JrPPO*2 core promoter region to contain jasmonic acid (JA)-, salicylic acid (SA)-, and ethylene (ERF)-responsive elements [27]. The presence of these biotic and abiotic stress-associated response factors explains the inducible nature of *JrPPO2* under stressful conditions, while their absence in the *JrPPO1* core promoter explains the differences in the expression patterns of these two genes. Analyses performed by other groups demonstrated similar increases in PPO activity during walnut blight, though no distinction was made between the expression or activities of the two walnut isoforms [28]. However, it is likely that the reported increase in PPO activity is due to induction of *JrPPO2* based on its described promoter features, with the noted genotypic variation in the degree of induction stemming from allelic variation within *J. regia*.

These findings confirm that *JrPPO2* acts as stress-activated defense enzyme, transforming diffusible phenolics into polymeric barriers. The quinones generated during this process are themselves antimicrobial, cross-linking microbial cell walls, denaturing enzymes, and sequestering essential metal ions. The localized pigment deposition is associated with increased resistance and restricted lesion expansion, implying a protective role for melanogenesis. Thus, the enzymatic browning long associated with plant injury represents a deliberate biochemical counterattack rather than incidental oxidation.

##### Interplay with Carbohydrate Metabolism and the SWEET Transport Network

Plant–pathogen interactions often involve metabolic tug-of-war over carbon resources. Pathogens attempt to redirect host sugars through induction of SWEET transporters, while hosts counter by restricting carbohydrate efflux. In walnut, genome-wide analysis identified 25 *SWEET* genes whose expression remained largely unchanged upon *Xanthomonas* infection, suggesting an alternative defensive allocation strategy [8]. Instead of restricting sugar flow, walnut appears to reallocate carbon into phenolic metabolism, fueling PPO-mediated oxidation and melanin formation as evidenced by the upregulation of *JrPPO2* and subsequent melanogenesis.

This redirection of metabolic flux underscores PPO’s role as a sink for excess reducing power during stress. By coupling phenolic oxidation to oxygen reduction, PPOs dissipate electrons that might otherwise feed pathogen respiration or generate damaging ROS. The interplay between SWEET transporter stability and PPO induction therefore exemplifies a metabolic pivot, from nutritional defense to oxidative fortification.

#### 4.4.3. Conceptual Implications for *JrPPO2*

Biochemically, *JrPPO2* acts as a redox amplifier, consuming molecular oxygen and generating quinones that polymerize into insoluble pigments, effectively creating oxidative barriers around damaged or infected cells. *JrPPO2* thus operates as a stress-responsive oxidase, transforming the oxidative burst into a structural and biochemical shield. The pigment barriers it forms not only limit pathogen spread but also prevent further ROS leakage, reinstating redox balance after the initial immune surge.

### 4.5. The Walnut Model for Plant Melanogenesis

Integrating the activities of *JrPPO1* and *JrPPO2* reveals a two-phase metabolic model for melanin formation in plants (Figure 1). In the first, “constitutive” phase, *JrPPO1* hydroxylates L-tyrosine to L-DOPA and regulates aromatic amino acid homeostasis, maintaining a pool of diphenolic intermediates within plastids. In the second, inducible phase, *JrPPO2* efficiently oxidizes these diphenols to o-quinones, which undergo non-enzymatic polymerization to yield melanin-like polymers within cells or in the apoplastic space. This stepwise process mirrors aspects of true melanin biosynthesis in animals, but with crucial distinctions: (i) substrate diversity, as plant PPOs act on a broad array of phenolics beyond those derived from tyrosine; (ii) compartmental segregation, preventing systemic oxidative damage; (iii) integration with phenylpropanoid metabolism and redox signaling. The end products, insoluble melanin-like polymers, exhibit the physicochemical properties of true melanin (light absorption, free radical scavenging, and redox buffering) but are chemically heterogenous, reflecting the diverse phenolic precursors available in plant cells.

Taken together, these findings establish a conceptual framework wherein *JrPPO1* and *JrPPO2* form a dual-enzyme system, orchestrating phenolic oxidation, redox homeostasis and defense associated pigmentation. This duality resolves a long-standing contradiction about the role of PPOs in plants: rather than serving exclusively as browning agents, they operate as metabolic sentinels, balancing basal detoxification and inducible defense. The resulting melanin-like polymers embody a biochemical compromise, sacrificing some phenolic carbon to generate an antioxidant, pathogen-resistant matrix that stabilizes cellular metabolism under stress. Unravelling this pathway not only demystifies plant melanogenesis but also opens new avenues for harnessing oxidative phenolic chemistry in crop improvement, postharvest management, and bio-based material design.

### 4.6. Systems-Level Integration: A Paradigm for Coordinated Oxidative Network

Comparative genomics of *Juglans* species reveal that the duplication and divergence of *PPO* genes represent a key evolutionary innovation [9]. The emergence of “constitutive” *JrPPO1* and inducible *JrPPO2* enabled walnut to partition oxidative metabolism into two specialized circuits: a housekeeping system that maintains phenolic balance and a responsive system that mobilizes pigment synthesis under stress. Across plant evolution, similar duplications have occurred independently in other lineages, supporting the notion that PPO diversification represents convergent evolution toward redox modularity. The capacity to control when and where melanin-like polymers form may have conferred a decisive advantage during colonization of oxygen-rich terrestrial environments.

Synthesizing this collective data from genomic, genetic, and phenotypic layers reveals a coherent systems-level model of oxidative metabolism underlying plant melanin formation:

- **Genomic control:** *PPO* loci, associated redox genes, as well as substrate-related regulatory and biosynthetic genes define the genetic potential for pigmentation.
- **Genetic activation:** Stress-regulated and developmentally regulated accumulation of PPO and oxidative enzymes executes pigment synthesis.
- **Phenotypic manifestation:** Variation in pigmentation intensity and pattern reflects the integrated outcome of enzyme activity, substrate composition, and redox balance.

This integration transforms walnuts from an isolated case study into a paradigmatic system for understanding oxidative polymerization in plants. This reveals melanogenesis to be an endpoint of cellular adaptation, bridging primary metabolism with long-term tissue resilience.

## 5. The Big Picture: PPOs as Mediators of Redox Homeostasis and Cellular Energetics

### 5.1. Phenolics Metabolism, Redox Homeostasis and Stress Response

As previously explored, silencing of *JrPPO1* revealed profound changes in tyrosine-derived metabolism [5]. Most notable of these changes was the accumulation of tyramine and hydroxycinnamoyl-tyramine conjugates. These changes suggest that *JrPPO1* has a role in the detoxification of tyramine, which is produced from tyrosine by the action of tyrosine decarboxylase. Tyramine itself demonstrates cytotoxicity when highly accumulated, and its conversion into hydroxycinnamoyl derivatives serves to both manage this toxicity and fuel other cellular processes such as cell wall reinforcement, defense against pathogens, nitrogen storage, and organ development [29]. As *JrPPO1* does not perform the function of tyrosine decarboxylase, the most plausible explanation for this distinct shift in metabolism is that, under normal conditions, intracellular tyramine levels are managed by *JrPPO1* through conversion to dopamine (monophenolase activity). Careful dissection of dopamine biosynthesis in purple false brome (*Brachypodium distachyon*) [30] has directly implicated plant PPOs in the metabolism of dopamine through this precise mechanism, and supports the hypothesis generated years ago in the walnut system. Thus, the loss of the PPO-mediated oxidative pathway does not merely alter phenolic composition but fundamentally destabilizes cellular redox balance through the disruption of tyramine homeostasis and dopamine biosynthesis. The resulting accumulation of tyramine and the consequent increase in reactive oxygen species, accumulation of electrophilic intermediates, and membrane damage collectively drive the lesion-mimicking phenotype and eventual cell death.

At the molecular level, PPOs act as redox valves within the broader metabolic circuitry. During stress, when photosynthetic or mitochondrial electron transport is over-reduced, PPO-mediated phenolic oxidation provides an alternate route for electron dissipation. These reactions occur most efficiently under milder acidic to neutral pH (typically pH 5.5–7.0), conditions characteristic of plastid compartments and the apoplast following cellular disruption. PPO activity also depends on the integrity of its CuA-CuB catalytic center, making copper availability and redox state critical determinants of enzyme function. The sensitivity of PPOs to metal chelation and redox active metals further underscores their role as redox regulated enzymes rather than simple pigment catalysts. By coupling substrate oxidation to oxygen reduction, PPOs drive excess reducing equivalents into melanin-like polymers, thereby preventing uncontrolled ROS generation.

This “sacrificial oxidation” transforms potentially harmful redox energy into a stable pigment matrix. The polymerization of quinones into melanin-like pigments is thus both a biochemical detoxification process and an energy-dissipating reaction. From a systems perspective, melanin formation represents an oxidative overflow pathway, analogous to photorespiration in chloroplasts, a controlled leakage route that preserves cellular stability when primary metabolism becomes saturated.

These PPO-mediated processes do not operate in isolation but intersect with the phenylpropanoid pathway and the many metabolic circuits that split off from it. In plants, phenylalanine, not tyrosine, is the precursor of many diverse groups of phenolic compounds by way of the phenylpropanoid pathway. Tyrosine instead contributes predominantly to specialized phenolic pools and to redox-active intermediates that feed PPO-dependent oxidation reactions Importantly, both phenylalanine and tyrosine are derived from the end-point of the shikimic acid pathway, arogenate, and thus indirectly impact each other and their derivatives. Consequently, as *JrPPO1* channels tyrosine-derived phenolics substrates into oxidative metabolism, it creates a metabolic sink that ultimately pulls carbon away from the phenylpropanoids and related pathways.

### 5.2. Functional Outcome: Polymerization as Protection

The culmination of this enzymatic and non-enzymatic cascade is deposition of a stable, redox-active polymer that confers physical and chemical protection. The polymer’s radical-scavenging capacity parallels that of eumelanin, suggesting convergent evolution toward protective redox buffering. Thus, the plant melanogenic process represents both a terminal step of phenolic catabolism and a durable record of the plant’s oxidative history, a biochemical scar tissue that fortifies the organism against further stress.

### 5.3. PPO as a Node Linking Defense and Programmed Cell Death

The trade-off between energy conservation and defense is managed through tightly regulated PPO activity. During pathogen challenge, PPO-mediated oxidation generates localized quinones that crosslink cell wall components and form physical barriers, while simultaneously limiting nutrient leakage [5]. These quinones also function as chemical deterrents, inactivating microbial enzymes and signaling the onset of programmed cell death (PCD).

The overlap between PPO activation and programmed cell death (PCD) suggests that PPOs mediate the transition from active defense to controlled sacrifice [5]. When infection or oxidative stress exceeds repair capacity, localized cell death confines the pathogen, and melanin-like deposition seals the damaged zone. PPO-derived quinones can covalently modify cysteine-rich proteins and transcription factors, potentially acting as redox-sensitive switches that trigger PCD. Conversely, as shown by *JrPPO1* silencing, the absence of PPO activity can also provoke runaway ROS accumulation and unregulated death (Section 4). Together these findings position PPOs as bidirectional modulators of plant immunity—either preventing or permitting cell death depending on metabolic context [5].

### 5.4. Ecological and Agronomic Consequences

In natural ecosystems, PPO-mediated pigmentation confers broad-spectrum protection against microbial decay and herbivory. Ultimately, PPOs exemplify the plant’s ability to convert biochemical danger into defensive structure: what begins as an oxidative burst ends as a layer of melanin-like armor. This transition from reactive chemistry to stable pigment encapsulates the evolutionary logic of the plant immune system, turning transient stress into lasting protection.

From an ecological and physiological standpoint, melanin deposition can be viewed as a signature of oxidative resilience. The polymer’s intrinsic properties, broad-spectrum UV absorption, metal chelation, and radical scavenging, extend the lifetime of exposed tissues, such as seed coats and bark, under fluctuating environmental conditions.

Similar adaptive pigmentation occurs in other perennial species, including coffee, tea, and apple, where PPO activity contributes to antioxidant capacity and pathogen resistance. These parallels suggest that plant “melanins” are functional analogs of the true melanins, divergent in chemistry but convergent in purpose, protection through oxidation.

#### 5.4.1. Commercial Relevance of *JrPPOs*: Walnut Fruit Browning

One of the most prominent contemporary challenges for walnut industries is the darkening of fruit tissues: for fresh walnuts it is the walnut hull (husk) and pellicle (seed coat), while for dried walnuts it is exclusively the pellicle. Darkening of these tissues leads to significant consumer rejection, leading to reduced profitability and increased food waste. The molecular mechanisms of these two phenotypes have consequently been the focus of much research to understand the factors that lead to and exacerbate their manifestation.

##### Postharvest Darkening of Fresh Walnut Fruit

In the case of fresh walnuts, PPOs have been implicated in the discoloration of both the green hull and pellicle. From a management perspective, PPO activity and tissue darkening can be mitigated through various postharvest technologies and combinations thereof. Low-temperature storage (LTS), low-O_2_-controlled atmosphere (CA), and application of PPO inhibitors all significantly preserve fresh walnut quality [31,32,33,34]. PPO inhibitors, such as ascorbic acid, have significantly reduced darkening in both hulls and pellicle, indicating mechanistic overlap between the darkening processes in these two tissues [33,34]. Interestingly, this overlap extends to the inclusion of ascorbic acid in plant tissue culture media which also prevented the browning of in vitro micro-propagated walnut explants: another PPO-mediated process that greatly mirrors the darkening observed during storage of fresh walnuts [35]. A deeper layer is revealed by experiments at modulating ethylene metabolism using 1-methylcyclopropene (1-MCP), which preserved hull and pellicle color when used in combination with LTS and CA [32]. Comparatively, treatment with the ethylene-producing agent ethephon (2-chloroethylphosphonic acid) produces a dose-dependent effect: with low doses of 10–500 mg/L providing quality retention while high doses of 8000 mg/L negatively impact quality [36]. Distinction between *JrPPO1* and *JrPPO2* were not made in these studies, but the presence of ethylene responsive elements in the promoter of *JrPPO2* suggests a mechanistic relationship between stress, ethylene signaling, PPO activity, and darkening. However, expression profiles for the two PPO isoforms vary significantly across studies, making it challenging to unify whether PPO induction is a hallmark of this darkening process.

While PPO activity and darkening are both suppressible through the aforementioned postharvest technologies, the most concrete link supporting the involvement of PPO in this process comes from genetics and functional genomics investigations. Gene silencing experiments utilizing a tobacco rattle virus (TRV)-based virus induced gene silencing (VIGS) approach of *JrPPO1* and *JrPPO2* in fresh walnut fruit resulted in significant and independent knock-down of their expression [37]. This knock-down in expression slowed the onset of hull browning, suggesting that both isoforms are involved in this process. Furthermore, these results suggest that both isoforms are being expressed in fresh walnut hulls during storage: likely coming from the basal expression of *JrPPO1* and stress-induced expression of *JrPPO2*. This was mechanistically corroborated by separate research endeavors which achieved a reduction in PPO activity and browning in the hull when applying transcriptional and translational inhibitory compounds, implicating active gene expression in the manifestation of darkening [38]. Taken together, these studies have revealed that PPO activity and consequent darkening of the fresh hull and pellicle during storage are not due, at least entirely, to the latently accumulated PPOs, but to their active expression. However, the unconfirmed melanic nature of the dark pigment produced in these processes leaves open the possibility that PPOs are not the only player, and that they may work in concert with other oxidative enzymes for pigment biosynthesis.

##### Postharvest Darkening of Dried Walnut Kernels

Separate from the darkening observed in fresh walnut fruits is the darkening associated with dried walnut kernels. Dried walnuts, both in-shell and shelled, are the predominant form of walnuts consumed worldwide owed in large part to their greater storability. Darkening that occurs exclusively in the thin pellicle, which is only observable after cracking, may be immediately observed or may occur gradually during subsequent post-harvest storage: indicating that this process spans both the production and post-harvest phases of walnut cultivation. However, unlike the discoloration that occurs in fresh walnuts, the mechanistic processes and underlying biochemistry of pellicle darkening during production and post-harvest storage of dried walnuts remain an open question.

The pellicle, like the hull, is rich in phenolic compounds and similar to fresh walnuts, LTS and CA (low O_2_) mitigate the loss of free phenolics, preserve antioxidant capacity, and delay darkening of dried kernels [39]. Similar effects are achieved by minimizing damage to the pellicle during shelling, which reduces the diffusion of oxygen into the pellicle at damaged sites that encourage phenolic oxidation [40]. The majority of these oxidizable pellicle phenolics are in a free form, rather than being esterified or bound [41]. Comparison of light and dark whole kernels of the cultivar ‘Chandler’ revealed lower total free phenolics and reduced antioxidant capacity in the dark samples [42]. Furthermore, a separate storage study of walnut kernels reported reductions in total phenols, flavonoids, and antioxidant capacity while kernel browning and PPO activity increased [43]. This suggests a framework in which extractable phenolics, which contribute to antioxidant capacity, are being utilized as precursors for the darkening reaction. More work is needed in this area to define the nature of the brown pigment and uncover the mechanisms behind its biosynthesis and accumulation.

While the previously discussed darkening during dried walnut storage is the predominant challenge for maintaining walnut quality, poor color at harvest due to darkening during production is a growing problem. Adverse climate events in major growing regions like California are known to negatively impact the initial color of dried kernels [44]. Metabolomics analysis of ‘Chandler’ kernels darkened at harvest indicated that dark pellicles have reduced free total phenolics and shed light on the species being depleted [44]. Among these were members of the flavanols, hinting that the formation of proanthocyanidins may be the underlying cause of darkening. However, the most interesting revelation comes from a separate comparison of light, medium, and dark walnuts at time of harvest [41]. This work revealed that while total free phenolics increased with lightness, flavanols were most enriched in the medium phenotype. This suggests that pellicle darkening in the field is an active biosynthetic process, where flavanols are triggered to accumulate and are then spent on pigment biosynthesis. Pre-harvest darkening of walnuts does not generally occur tree-wide but is rather localized to individual fruit, with stress events triggering pellicle darkening. The pellicle at this point is a moist, metabolically active tissue, and stress-induced biosynthesis of secondary metabolites, like the flavanols, is consistent with the tissues’ role in seed protection. Comparatively, post-harvest darkening of dried kernels is likely not an active biosynthetic process but rather utilizes the pools of flavanol substrates established during production which are slowly converted into similar colored pigments through currently unknown, though potentially enzyme-mediated, mechanisms.

### 5.5. Implications for Crop Improvement and Functional Genomics

The convergence of multi-omics evidence provides not only mechanistic clarity but also translational opportunity. Breeding or engineering approaches that fine-tune PPO expression, localization, or activity could modulate pigmentation and enhance oxidative stress tolerance. Moreover, the identification of co-regulated pathways offers candidate targets for manipulating redox buffering capacity without compromising defense function.

In a broader sense, these studies exemplify how integrating omics data can expose the molecular architecture of enigmatic plant pathways, resolving decades-old biochemical questions through genome-enabled precision.

### 5.6. A Unified Model of the Plant Melanin Pathway

Integrating biochemical, genetic, and evolutionary insights yields a unifying model:

1. **Constitutive phase:** *JrPPO1* continuously hydroxylates tyrosine, sustaining phenolic homeostasis and preventing accumulation of reactive amines.
2. **Inducible phase:** *JrPPO2* activates under stress, oxidizing phenolics into quinones that polymerize into melanin-like pigments.
3. **Protective outcome:** The resulting polymer sequesters reactive oxygen and metals, forming a barrier that stabilizes cellular redox potential.
4. **Evolutionary optimization:** Gene duplication and regulatory specialization of PPOs allow flexible allocation between growth and defense across environmental contexts.

In this framework, melanin biosynthesis is neither vestigial nor incidental, it is a strategic, energy-intensive adaptation that enables plants to survive and reproduce in oxidative environments. The walnut system provides the clearest experimental demonstration of this principle, converting what was once a biochemical curiosity into a model for adaptive oxidative metabolism.

## 6. Conclusions and Perspectives

Despite decades of study, melanin biosynthesis in plants remains one of the most intriguing and unresolved enigmas of plant biochemistry. The work on walnut (*Juglans regia*) has begun to lift this veil, revealing that plant melanin formation is neither incidental nor artifactual but rather an evolved oxidative program integrated within the plant’s metabolic and defense networks. At the center of this program lies the dual polyphenol oxidase (PPO) system, the constitutive *JrPPO1* and the inducible *JrPPO2*—that together independently catalyze the transformation of tyrosine and related phenolics into melanin-like polymers. These enzymes embody a remarkable metabolic flexibility: under normal growth, *JrPPO1* maintains aromatic amino-acid homeostasis and phenolic redox balance; under stress, *JrPPO2* mobilizes oxidative polymerization to seal wounds, neutralize radicals, and reinforce cellular boundaries. Their interplay converts the transient chemistry of phenolic oxidation into a controlled and compartmentalized defense mechanism, protecting tissues from oxidative collapse while simultaneously recording the plant’s redox history in a dark, protective pigment. The walnut model reveals that plant “melanin” is not a single molecular entity, but a heterogeneous ensemble of oxidized phenolic polymers produced through enzyme-initiated yet chemically self-propagating reactions. These pigments differ structurally from the true melanins but converge functionally, serving as broad-spectrum antioxidants, UV screens, and antimicrobial barriers. Their synthesis is costly in terms of carbon and reducing power, yet the conservation of PPOs across diverse plant lineages underscores the adaptive value of this oxidative expenditure. Melanogenesis in plants thus represents a trade-off between defense and metabolism—an oxidative insurance policy against environmental uncertainty.

A number of key questions remain open. The exact chemical architecture of plant melanins, how various phenolic building blocks co-polymerize and organize into functional aggregates, remains to be resolved through advanced spectroscopic and mass-spectrometric approaches. The regulatory hierarchy that coordinates PPO activation with redox signaling, calcium flux, and stress hormones also requires elucidation. Moreover, the ecological and physiological contexts that determine whether PPO activity results in beneficial protection or deleterious darkening are only beginning to be understood. Addressing these gaps will require the integration of metabolomics, spatial proteomics, and in vivo imaging to capture the dynamics of oxidative polymerization at cellular resolution. Beyond fundamental insight, these discoveries open translational frontiers. Engineering PPO expression or subcellular targeting offers a means to modulate pigment formation, enhance stress tolerance, and improve postharvest stability in crops. Controlled melanogenesis could yield natural antioxidant coatings, UV-protective films, or bio-composite materials derived from plant tissues. The walnut system, amenable to genetic manipulation and rich in phenolic precursors, provides a unique experimental and biotechnological platform to explore such innovations. In summary, unraveling walnut’s “dark secret” has reframed plant melanogenesis as a dynamic, redox-regulated adaptation rather than a byproduct of decay. The convergence of genomic, genetic, and physiological evidence portrays PPOs as metabolic sentinels balancing energy, defense, and survival. As this field advances, what once appeared as mere darkening may emerge as one of nature’s most elegant oxidative designs—this is a testament to the way in which plants transform chemical stress into structural resilience through the art of controlled darkness.

## Data Availability

No new data were created or analyzed in this study. Data sharing is not applicable to this article.

## Abbreviations

The following abbreviations are used in this manuscript:

PPO	polyphenol oxidase
ROS	reactive oxygen species
EB	enzymatic browning
MALDI-TOF MS	matrix-assisted laser desorption ionization–time of flight mass spectrometry
CuA	copper-binding domain A
CuB	copper-binding domain B
L-DOPA	L-3,4-dihydroxyphenylalanine
Jr	*Juglans regia*
MYA	million years ago
Aa	amino acid
NADPH	nicotinamide adenine dinucleotide phosphate
JA	jasmonic acid
SA	salicylic acid
ERF	ethylene response factor
LTS	low-temperature storage
CA	controlled atmosphere
1-MCP	1-methylcyclopropene
